# Microplastics pollution in salt pans from the Maheshkhali Channel, Bangladesh

**DOI:** 10.1038/s41598-021-02457-y

**Published:** 2021-11-30

**Authors:** Md. Refat Jahan Rakib, Sultan Al Nahian, María B. Alfonso, Mayeen Uddin Khandaker, Christian Ebere Enyoh, Fauziah Shahul Hamid, Abdullah Alsubaie, Abdulraheem S. A. Almalki, D. A. Bradley, Hamidreza Mohafez, Mohammad Aminul Islam

**Affiliations:** 1 Department of Fisheries and Marine Science, Faculty of Science, Noakhali Science and Technology University, Noakhali, Bangladesh; 2 Bangladesh Oceanographic Research Institute, Cox’s Bazar, Bangladesh; 3 Instituto Argentino de Oceanografía (IADO-CONICET-UNS), Florida 8000, B8000BFW Bahía Blanca, Argentina; 4 Centre for Applied Physics and Radiation Technologies, School of Engineering and Technology, Sunway University, 47500 Bandar Sunway, Selangor Malaysia; 5 Group Research in Analytical Chemistry, Environment and Climate Change (GRACE & CC), Department of Chemistry, Faculty of Science, Imo State University Owerri, P. M. B 2000, Imo State, Nigeria; 6 Institute of Biological Sciences, Faculty of Science, University of Malaya, 50603 Kuala Lumpur, Malaysia; 7 Center for Research in Waste Management, Faculty of Science, University of Malaya, 50603 Kuala Lumpur, Malaysia; 8 Department of Physics, College of Khurma, Taif University, P.O. Box 11099, Taif, 21944 Saudi Arabia; 9 Department of Chemistry, Faculty of Science, Taif University, Taif, 21974 Saudi Arabia; 10 Department of Physics, University of Surrey, Guildford, GU2 7XH UK; 11 Department of Biomedical Engineering, Faculty of Engineering, Universiti Malaya, 50603 Kuala Lumpur, Malaysia; 12 Department of Electrical Engineering, Faculty of Engineering, Universiti Malaya, 50603 Kuala Lumpur, Malaysia

**Keywords:** Biochemistry, Cancer, Chemical biology, Ecology, Molecular biology, Biogeochemistry, Environmental sciences, Environmental social sciences, Natural hazards, Ocean sciences, Solid Earth sciences, Cardiology, Diseases, Health care

## Abstract

Microplastics (MP) were recognized as an emergent pollution problem due to their ubiquitous nature and bioaccumulative potential. Those present in salt for consumption could represent a human exposure route through dietary uptake. The current study, conducted in Bangladesh, reports microplastics contamination in coarse salt prepared for human consumption. Sea salt samples were collected from eight representative salt pans located in the country's largest salt farming area, in the Maheshkhali Channel, along the Bay of Bengal. Microplastics were detected in all samples, with mean concentrations ranging from 78 ± 9.33 to 137 ± 21.70 particles kg^−1^, mostly white and ranging in size from 500–1000 µm. The prevalent types were: fragments (48%) > films (22%) > fibers (15%) > granules and lines (both 9%). Fourier transform mid-IR and near-IR spectra (FT-MIR-NIR) analysis registered terephthalate (48%), polypropylene (20%), polyethylene (17%), and polystyrene (15%) in all samples. These results contribute to the MP's pollution knowledge in sea salts to understand and reduce this significant human exposure route and environmental pollution source in the future.

## Introduction

Plastic pollution in the marine environment has become a major global problem^1^. The degradation of the plastic waste in the seawater is not the end of the problem as the microplastics (MP) generated have a destructive potential for the environment. Added to this is the release of chemical compounds, further worsening the damage to the environment^2^. Furthermore, most plastics in the environment endure for a very long time^3^. According to the G20 Implementation Framework for Actions on Marine Plastic Litter, 20 countries are responsible for 80% of the plastic debris found in the sea, from which 90% originate from land-based sources. According to a study by Jambeck et al.^4^, the significant percentage of the world's ocean plastics pollution is from Asia, with China contributing 28% of the mismanaged plastic waste, followed by Indonesia (10%), the Philippines and Vietnam (both 6%), Thailand 3.2%, Egypt 3%, Nigeria 2.7%, and South Africa 2%.

MPs become more threatening than large plastic materials because they could be swallowed and concentrated by aquatic organisms, including plankton^5, 6^. A lower limit to MP size study has not yet been defined, although most investigations have focused on the 0.3–5 mm size range^7^. MP can be classified into two basic groups, namely primary and secondary, depending on their origin. Primary MP are industry-made particles, mainly used in commercial formulations, from cosmetics and toothpaste to micro-additives in synthetic paints and coatings^8^. Secondary MP results from the fragmentation of larger plastics^8^.

When it comes to MP pollution, salts for consumption are not exempt from the problem. Table salts are obtained from mining mineral rock or the evaporation of water sources at sea^1^. During the production process, saltwater undergoes different physical processes. It is first pumped into evaporation ponds, subsequently concentrated and crystallized by the action of the sun and wind, then being cut and packed for sale^9^. Accordingly, the final product may contain concentrates of the anthropogenic contaminants already present in the saltwater^10^.

Sea salt is highly commercialized, being consumed worldwide, with the number of studies focusing on MP pollution in table salts increasing^11, 12^. Around 60 salt processing mills exist in the Cox's Bazar district in Bangladesh^9^, wherein washing, crushing, iodine mixing, drying, and packing are carried out. About 25% of the raw salt is transformed as waste during processing, while the remaining 75% is crushed and packed as iodide salt and distributed throughout the country, predominantly for human consumption. Despite the high consumption rates, there has been a lack of data on the presence of MP in table salt from Bangladesh. Therefore the present study seeks to analyze for the first time the abundance, characteristics, and polymer composition of MP pollution in commercial salts obtained from salt pans along the Bangladesh coast. It is expected that this study will form a baseline for MP salt pollution for the country, also enhancing knowledge about this emergent pollutant issue.

## Material and methods

### Study area

**Figure 1:**
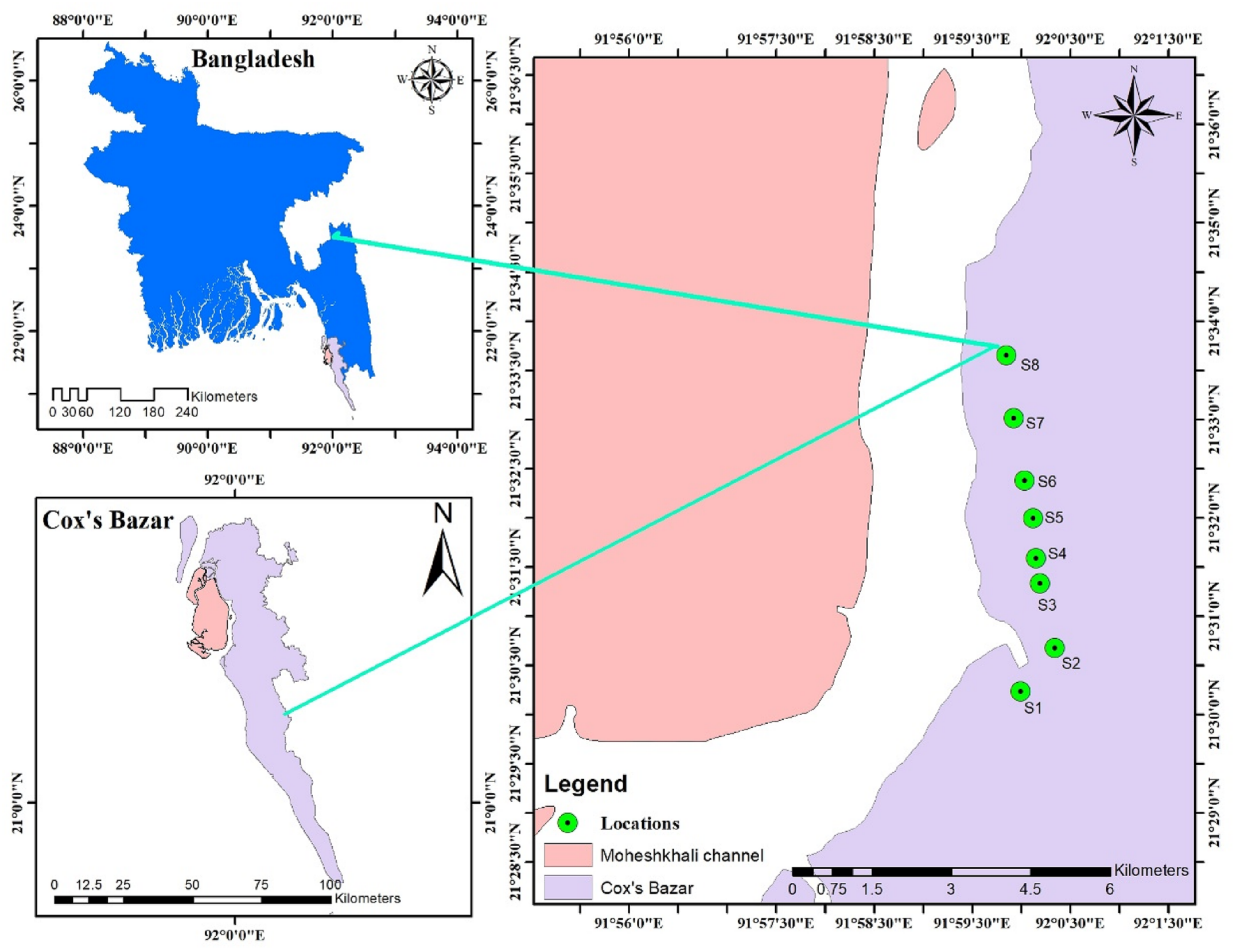
The geographic location of the sampling points along the Maheshkhali Channel coast. This map was constructed using ArcGIS 10.7.

Located at the head of the Bay of Bengal, the coastline of Bangladesh represents a diversified hydro-geo-morphological environment, with several interruptions by estuarine inlets^13^. The present study was performed in the Maheshkhali Channel (MC), located on the southeast coast of the Bay of Bengal (Fig. 1). The channel includes the Bakkhali River to the south, discharging domestic, agricultural, and industrial waste before falling into the Bay^14, 15^. This area has a semidiurnal tidal regime, with sea currents increasing from south to north, mainly near the coastal area^16^. Its hydrology is also heavily influenced by the monsoon season (June–September)^9^. The surrounding area to MC is highly urbanized, including Chittagong in the north, forming the second-largest city in Bangladesh (approaching 8,440,000 inhabitants) and Cox's Bazar beach at the east (with 85,000 daily visitors) (Fig. 1), the longest natural beach in the world with many hotels, a fishing industry, tourism and unplanned urbanization (e.g., a camp for displaced Rohingya refugees)^9^.

The MC is one of the most popular places in Bangladesh for salt production^9^. Extended mud flat shores are present along its coast, where most salt pans have been established^9^. The post-monsoon season (November to April) gives rise to favorable meteorological conditions for salt production, farmers selling the raw salt directly to traders^9^. Besides salt pans and processing industries, other productive activities developed in this area are traditional fisheries, commercial shrimp farms, a fishing port, fish processing industries, ship and boat making factories, and paint industries. Therefore, MC is seriously impacted by chemical effluents and waste effluents from many unplanned industries^14^.

### Samples collection

A total of eight representative salt pans (large in terms of relative size or hosting the greatest numbers of salt pans in a particular location) were selected to collect the samples for this study. Unrefined sea salt for consumption samples were collected from the selected natural salt pans along the southeast coast of MC (S1-S8) (Fig. 1). Sampling was performed between August (2020) and September (2021) (the post-monsoon period). Approximately 500 g of salts were collected using a metal spoon at each site, placed in a clean labeled 1 L glass bottle, and transported to the laboratory.

### Samples digestion and treatment

**Figure 2:**
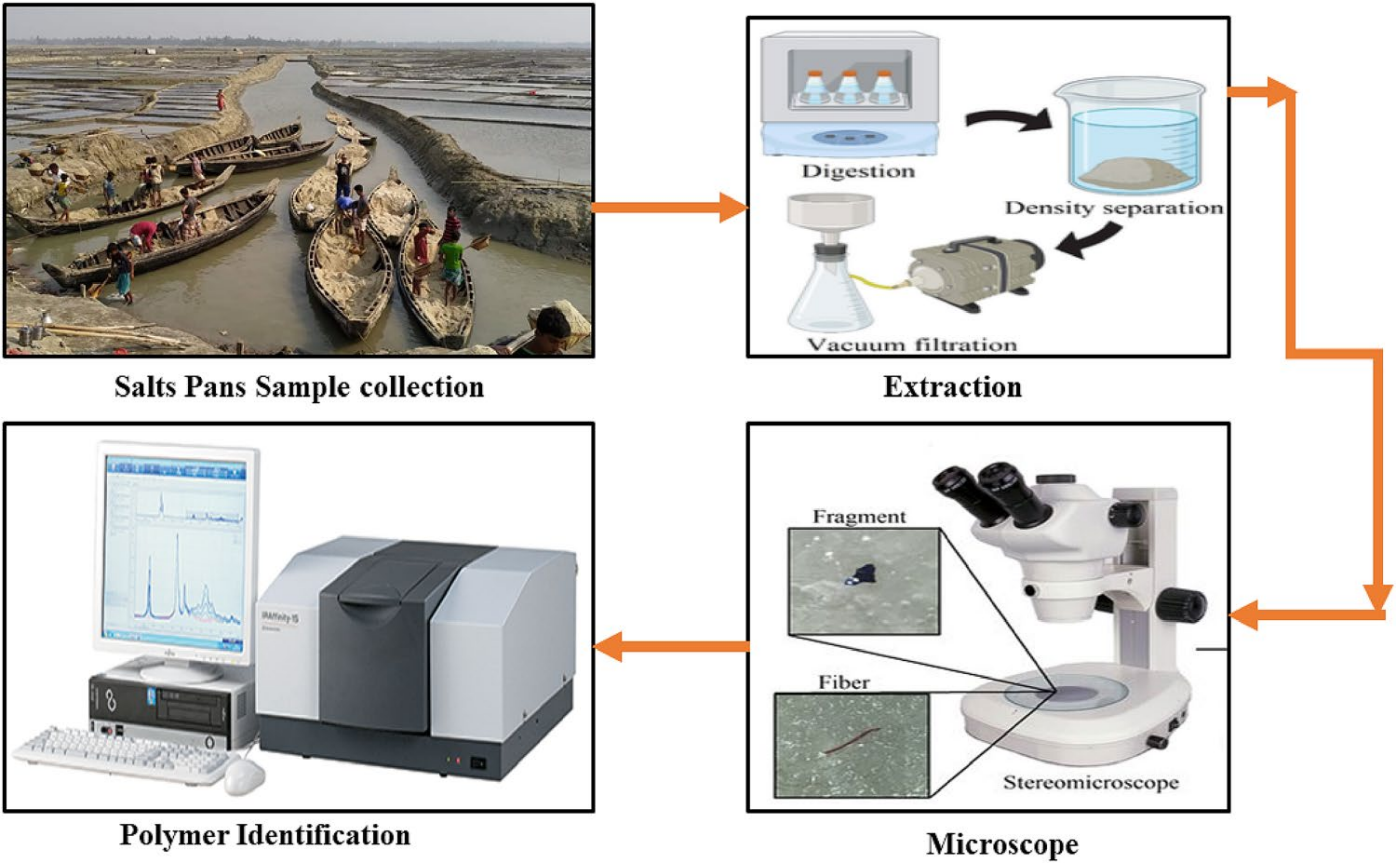
Procedural steps for microplastics analysis in sea salts samples. This diagram was constructed using PowerPoint 2016.

Each sample was divided into three (n = 3) subsamples for further analyses. Then 100 mL of 30% H_2_O_2_ were added to each sample in cleaned glass flasks and heated in a bath held at 65 °C for 24 h to digest any organic matter (Fig. 2). Approximately 1000 mL of filtered water was added to each bottle, and a glass rod was used to stir the salts until fully dissolved. The obtained salt solution was immediately filtered with cellulose nitrate filter paper (47 mm diameter, 0.45 μm pore size) using a vacuum system. The filter was then placed into a clean glass Petri dish and dried at room temperature.

### Quality control

All liquids were filtered using 0.45 μm pore size filter paper before use to avoid MP contamination. Also, all the glass material was rinsed three times with purified water. The samples were kept covered when they were not under analysis. Lab staff used cotton lab coats and nitrile gloves to avoid any source of extraneous plastic contamination. Three blank samples without salt were analyzed simultaneously to correct for any possible MP contamination from sample processing. No MP were found in blank samples.

### Visual observation and polymer FT-MIR-NIR analysis

The filters were visually inspected using an Olympus SZX16 stereomicroscope, and images were taken. Visual assessment was performed in order to identify the shape and color of MP. Then, particles were randomly selected from each site (approximately 30%) for Fourier Transform Mid- and Near-Infra Red (FT-MIR-NIR) polymer analysis using a Perkin Elmer FT-MIR-NIR system. MP abundance was calculated based on visual observation and FT-MIR-NIR plastic polymer confirmation. For FT-MIR-NIR analysis, the spectrum range was set to 4000–675 cm^−1^ with a 3 s and 8 cm^−1^ resolution collection for all samples. All the spectra were then compared with the spectra library to identify the polymer type.

### Data analysis and risks assessment

Statistical analysis was performed using SPSS Version 20 (IBM®). Results were reported as mean values and standard deviation from replicates. Due to the non-normal distribution of data, the Kruskal–Wallis test followed by pairwise Dunn's tests were performed to determine any differences in MP concentrations among sampling sites^17^. Results were presented as boxplot and p-values. Statistical significance was set at p < 0.05.

#### MP risks assessment

Besides the residual monomers, additional polymerization pollutants may be present in a plastic object. These include oligomers, low-atomic-weight polymer segments, catalyst residues, and polymerization solvents, as well as a broad range of plastic additives, such as handling aids and final product additives^18^. Because the majority of these non-polymeric segments have a low atomic weight, they could migrate from the MP item into the salt and then into humans, where they may cause damage. Therefore, it is crucial to estimate the risks of these polymers. The risks assessment of MP in the salt were estimated using the polymer risk indices (pR_i_) following the procedure detailed in previous work^18^, presented in Eqs. (1) and (2) as follows:

1 $${pR}_{i}=\sum \left(\frac{{p}_{i}}{{p}_{T}} \times {S}_{j}\right)$$

2 $${pR}_{overall}=({pR}_{1}\times {pR}_{2}\times {pR}_{3}\times \dots {pR}_{n}{)}^{1/n}$$

where p_i_ is the number of each MP polymer observed in salt sample i and p_T_ is the total number of different MP polymers identified in salt sample i. S_j_ is the chemical toxicity coefficient or risk score, given as Polyethylene (PE), 11; Polyethylene terephthalate (PET), 4; Polypropylene (PP), 1, and Polystyrene (PS), 30^18^. The polymeric risk indices (pRi) classification is as follows: low when < 150, medium when 150–300, considerable when 300–600, high 600–1200, and very high when > 1200^18^. Due to the simplicity of the index, it could be applied to different types of environments, but at the same time, the conclusions from it are limited and should be considered with caution.

## Result and discussion

### MPs abundance

**Figure 3:**
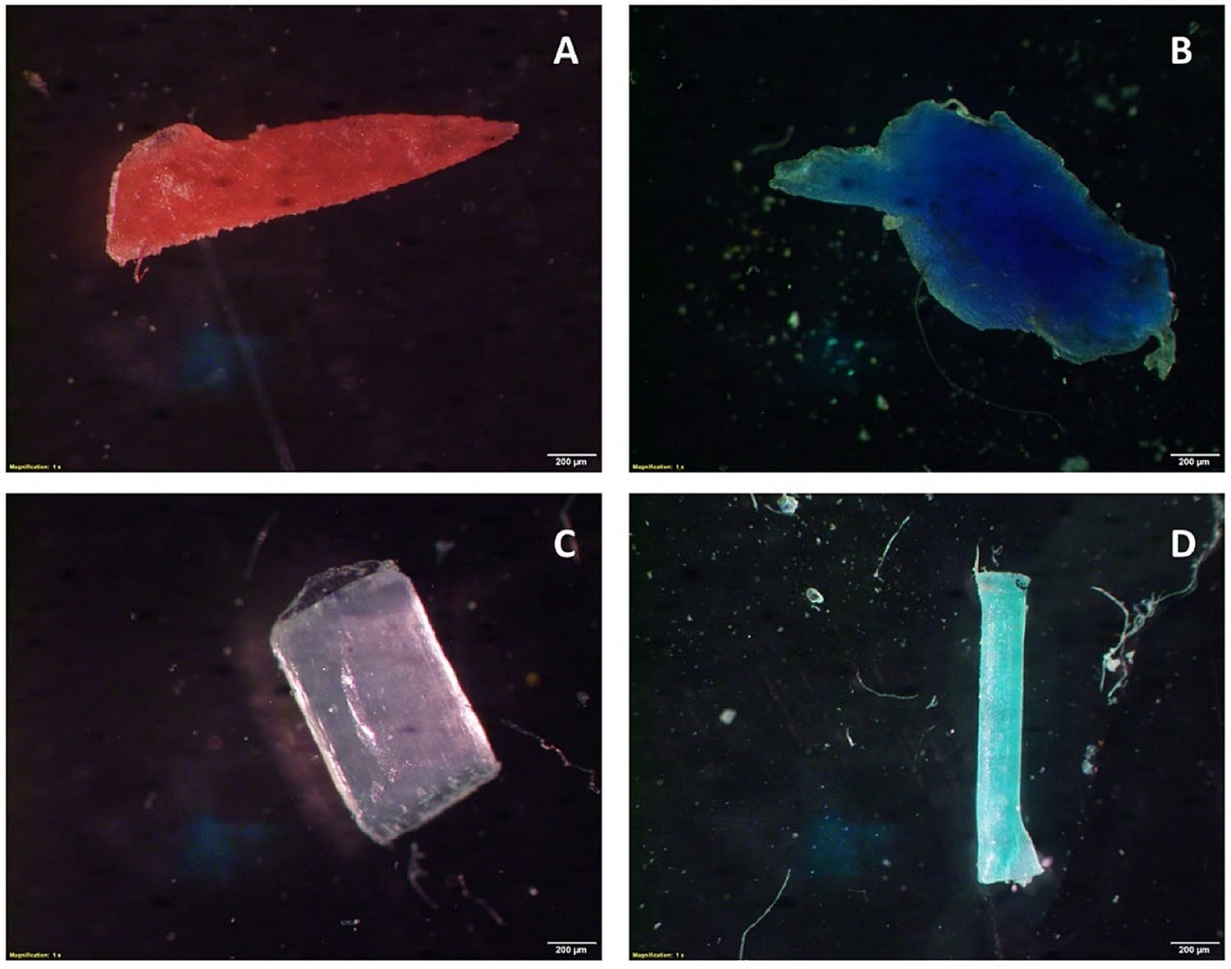
Photographs of different MP shapes found in salt samples: (**a**) red fragment; (**b**) blue fragment; (**c**) pellet; (**d**) line.

**Figure 4:**
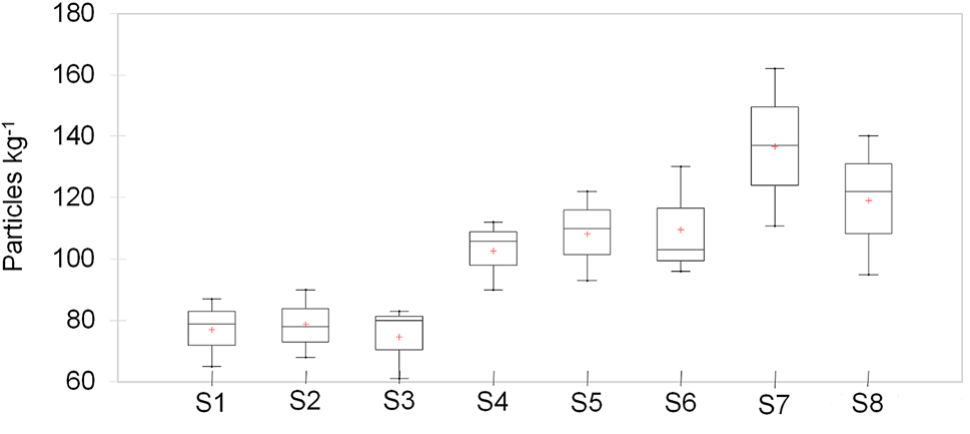
Box plot of MP abundance (particles kg^−1^) for the sampling sites S1 to S8.

In Table 1 MP abundance (mean value ± standard deviation) values are presented by shape, size range, color and polymer type categories for each sampling site. MP were found in all analyzed salt samples including pellets, fibers, fragments, films and lines (Fig. 3). MP total abundance values per site ranged from 74.7 to 136.7 particles kg^−1^ in the following order of increasing abundance: S3 < S1 < S2 < S4 < S5 < S6 < S8 < S7 (Fig. 4, Table 1). The measured values show significant difference among sites (Kruskal Wallis, p = 0.013). The Dunn’s test results reveal significant differences, with S1, S2 and S3 differing from S7 (p = 0.006; p = 0.007 and p = 0.005, respectively) and S8 (p = 0.023; p = 0.028 and p = 0.019, respectively), S3 also being significantly different from S6 (p = 0.05).

**Table 1:** Microplastic abundance (Particles kg^−1^) (Mean value ± SD) by shape, color, size range and, polymer categories in unpacked coarse salt samples from stations S1 to S8 (n = 3).

	S1	S2	S3	S4	S5	S6	S7	S8
**Shape**								
Pellet	6.3 ± 2.1	8.7 ± 3.5	10.7 ± 2.3	8.0 ± 2.0	7.0 ± 3.0	12.7 ± 4.0	8.7 ± 2.1	9.3 ± 6.4
Fiber	5.3 ± 1.5	8.7 ± 3.0	14.0 ± 4.6	18.3 ± 3.0	12.3 ± 3.5	18.0 ± 3.6	26.3 ± 5.0	19.7 ± 5.7
Fragment	37.7 ± 3.5	43.7 ± 9.1	27.0 ± 9.2	42.7 ± 10.5	32.7 ± 4.7	51.3 ± 13.6	59.3 ± 16.9	65.3 ± 7.5
Film	20.3 ± 6.1	13.7 ± 6.6	15.7 ± 5.5	22.7 ± 5.7	39.3 ± 7.5	22.0 ± 3.6	30.3 ± 5.5	17.7 ± 6.1
Line	7.3 ± 3.5	4.0 ± 2.0	7.3 ± 1.5	11.0 ± 2.0	17.0 ± 5.3	5.7 ± 2.5	12.0 ± 5.0	7.0 ± 2.6
**Color**								
White	8.0 ± 3.6	10.3 ± 2.5	7.3 ± 3.1	10.7 ± 1.5	9.7 ± 2.1	11.3 ± 5.0	19.7 ± 4.2	14.0 ± 5.3
Blue	2.3 ± 0.6	3.0 ± 1.0	6.3 ± 1.5	5.3 ± 2.5	4.0 ± 2.0	6.3 ± 1.5	5.7 ± 1.5	4.3 ± 2.5
Green	3.3 ± 0.6	1.7 ± 0.6	2.3 ± 2.1	3.0 ± 2.6	3.3 ± 1.5	5.3 ± 4.2	3.7 ± 2.5	2.7 ± 1.2
Black	4.3 ± 1.5	4.3 ± 1.5	3.0 ± 1.7	5.0 ± 2.6	5.7 ± 3.1	3.0 ± 2.0	7.0 ± 5.2	9.0 ± 3.6
Pink	1.7 ± 0.6	1.0 ± 1.0	0.7 ± 0.6	4.0 ± 1.0	2.7 ± 1.5	2.0 ± 1.0	1.3 ± 0.6	1.0 ± 1.0
Transparent	2.3 ± 0.6	2.7 ± 0.1	2.3 ± 0.6	1.7 ± 1.5	3.7 ± 3.1	3.0 ± 1.0	3.3 ± 1.5	4.7 ± 2.1
Colorless	1.3 ± 0.6	2.0 ± 1.0	0.7 ± 0.6	1.0 ± 1.0	2.7 ± 0.6	3.7 ± 1.5	1.0 ± 1.0	1.7 ± 0.6
**Size range (μm)**								
250–500	6.0 ± 3.0	7.0 ± 2.6	6.3 ± 3.1	8.7 ± 4.7	6.7 ± 4.2	9.0 ± 2.6	10.7 ± 4.0	9.0 ± 2.6
1000–500	9.7 ± 2.5	10.3 ± 4.2	7.0 ± 2.6	12.3 ± 2.1	13.0 ± 4.0	13.7 ± 4.2	17.3 ± 5.5	15.3 ± 2.5
1000–5000	7.7 ± 3.1	7.7 ± 2.5	9.3 ± 3.1	9.7 ± 2.1	11.0 ± 7.2	12.0 ± 3.0	13.7 ± 5.7	13.0 ± 5.3
**Polymer type**								
PP	7.0 ± 4.4	5.3 ± 2.08	5.3 ± 2.1	6.3 ± 1.5	7.7 ± 3.2	7.0 ± 4.0	9.3 ± 3.1	7.0 ± 2.0
PET	10.7 ± 4.2	11.7 ± 3.1	10.0 ± 6.2	15.3 ± 4.2	17.3 ± 4.9	19.3 ± 7.8	22.0 ± 6.6	21.7 ± 9.1
PS	4.7 ± 1.5	4.3 ± 1.5	5.0 ± 4.6	5.0 ± 2.0	5.0 ± 4.4	5.7 ± 3.1	5.7 ± 3.8	5.0 ± 1.7
PE	3.7 ± 2.1	5.0 ± 1.0	4.7 ± 1.5	7.3 ± 4.2	6.3 ± 6.7	4.3 ± 2.1	8.7 ± 3.1	6.0 ± 1.0
Total	77.0 ± 11.1	78.7 ± 11.0	74.7 ± 11.9	102.7 ± 11.4	108.3 ± 14.6	109.7 ± 17.9	136.7 ± 23.5	119.0 ± 22.6

It is expected that MP in seawater was the primary source of contamination of the sea salts^19–22^. According to these results, MP contamination in the salt pans from the Maheshkhali Channel coast can be classified into three zones of increasing pollution: a lower zone (S1 to S3), an intermediate zone (S4 and S5), and a higher zone (S6 to S8). A possible explanation for these values could be the different disposition of the study sites in the channel and the hydrology and current (rate of water movement) differences. According to Misra et al.^16^, seawater current flows with higher intensity from the south of the Bay of Bengal to the north. Therefore, any MP in seawater flowing from the Bay of Bengal inside MC (S1 to S8 direction) will probably accumulate in the inner part of the channel. However, the higher MP concentration in S7 with respect to S8 suggests that other environmental variables or anthropogenic sources could affect MP presence in MC, and more variables should be assessed in future studies to arrive at better conclusions. It should also be mentioned that the plastic film used in salt pans for desiccation is of high potential as a source of MP. Other possible MP sources could be plastic pollution from fishing, urbanization, and tourism activities in the surrounding area. Furthermore, runoff from the land and atmospheric fallout could also be potential contributory pathways^23^, not least given that Bangladesh is subject to the influence of the monsoon, with high rain values and salt production developing after the monsoon season^9^. Future research, including seawater and atmospheric MP samples, should be considered to confirm these assumptions.

Although there are no previous studies of MP abundance in salt samples, other studies have been conducted in the coastal zone of the Bay of Bengal adjacent to Bangladesh. A study conducted by Rahman et al.^24^ in beach sediments from Cox's Bazar beach registered relatively low MP values of 8.1 ± 2.9 particles kg^−1^ while, in a study of intertidal sediments from the same area by Hossain et al.^25^, higher MP values of 368.68 ± 10.65 particles kg^−1^ were reported. Both studies attributed their spatial MP variation to be due to the tidal current, wave energy, beach orientation, river discharges, and human activity. Finally, another study in beach sediments found MP concentrations up to 1100 particles kg^−1^, attributing these high values to increasing urbanization and tourism^26^.

The results obtained in this study have been compared with other salt studies worldwide (Table 2). However, it should be mentioned that most studies were developed with refined commercial sea salt samples and not from field salt pans as in this case. In addition, the different analytical methods used for MP determination difficult results comparison. The MP concentrations found in the salts from MC are similar to those reported by studies in Brazil, Mexico, South Korea, and Indonesia^12, 27^ (Table 2). On the other hand, studies of salt samples from the Atlantic and Indian Oceans^12^, the Pacific Ocean (China and Thailand)^19^, and the Mediterranean Sea (Croatia, Italy)^27, 28^ presented higher MP concentrations (Table 2). These studies detected the presence of MP of smaller size than those registered here, supporting the observation of the higher values. It could be expected that the fragmentation of MP particles during salt processing for commercial salts could also be contributing to the increasing number of particles found in salt samples.

**Table 2:** Comparison of present and worldwide values for microplastics abundance, size range, and polymer types in commercial sea salt samples.

Sea	Country	MP characteristics			References
		Polymer type	Particle size range (μm)	Concentration (Particles kg^-1^)	
Bay of Bengal	Bangladesh	PP, PET, PS, PE	250–5000	78–137	This study
Pacific Ocean	Thailand	PE, PET, PP, PVC	100–5000	80–600	^12^
	Vietnam	Acrylic, PE, PP, PU	100–5000	100–200	^12^
	New Zealand	PE	160–980	0–1	^11^
	South Korea	Acrylic, Nylon, PE, PET, PP	100–3000	100–300	^12^
	Japan	PE, PET	160–980	0–1	^11^
	Chinese Taipei	PE, PET, PP	100–2000	0–1300	^12^
	Australia	Acrylic, Nylon, PE, PET, PP, PS	100–3000	80	^12^
Mediterranean Sea	Turkey	PE, PET, PP, PU, PA, PVC	< 100	18–84	^28^
	Spain	PE, PET, PP	30–3500	80–280	^29^
	Italy	PE, PP	4–2100	22–594	^27^
	France	PE, PET, PP	160–980	0–2	^11^
	Croatia	PE, PP	15–4628	13,500–19,800	^27^
Indic Ocean	India	Nylon, PE, PET, PP, PVC	50–600	100–5000	^12^
	Malaysia	PP	160–980	0–1	^11^
Black Sea	Bulgaria	Nylon, PE, PP, PVC	100–4000	10	^12^
Atlantic Ocean	Brazil	PET, PP	100–1000	200	^12^
	Portugal	PET, PP	160–980	0–10	^11^
	Senegal	PE, PET, PP	100–3000	250	^12^
	South Africa	PET	160–980	1–3	^11^
	United Kingdom	PP, PE, PVC	100–2000	120	^12^
	USA	PE	100–1000	3000	^12^

*PET* Polyethylene terephthalate, *PP* Polypropylene, *PE* Polyethylene, *PVC* Polyvinyl chloride, *PS* Polystyrene, *PA* Polyamide, *PU* Polyurethane.

### MP shape, size, and color

**Figure 5:**
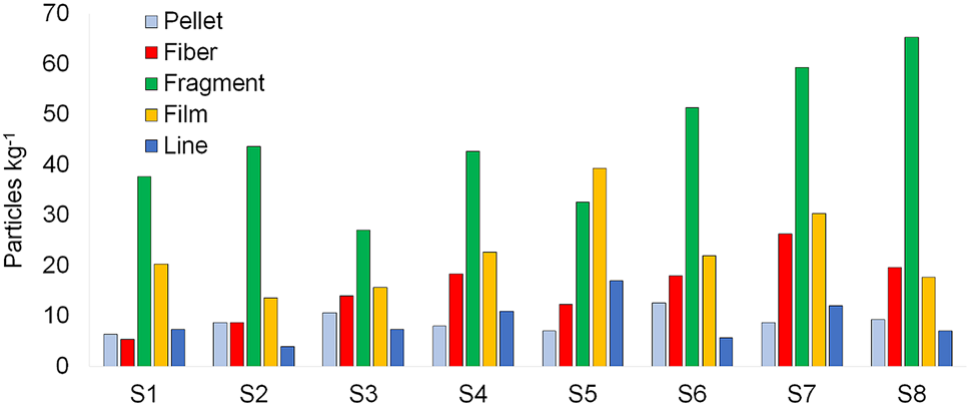
Microplastics abundance (particles kg^−1^) by shape category registered at the sampling sites S1 to S8.

The fragment and film MP categories were the most abundant shape types (Table 1, Fig. 5), coinciding with the results reported for sea salts samples worldwide^12^. The order of distribution based on MP shape was: fragments (48%) > films (22%) > fibers (15%) > pellets and lines (both 9%). Higher quantities of fragments and films were also reported for Indian salts samples^20^. The studies analyzing MP in Cox's Bazar sediments registered fibers and fragments dominating shape composition^15, 23–26^. Given that this area is highly touristic and urbanized, plastic fibers from clothes and fabrics could be a major source.

**Figure 6:**
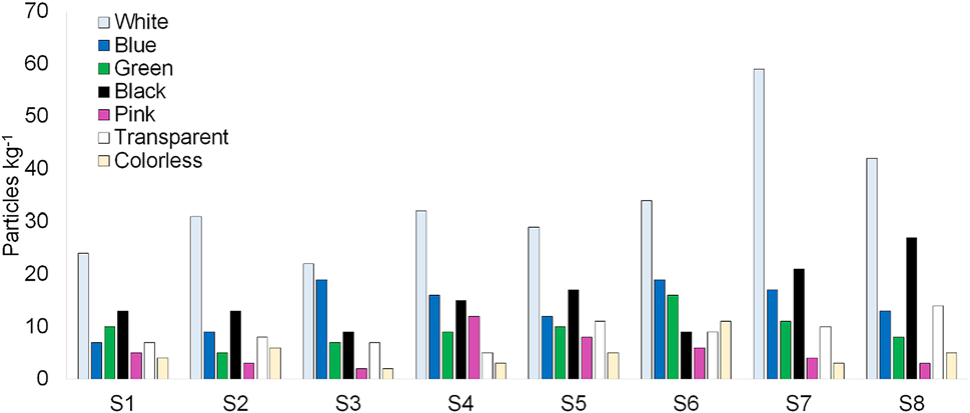
Microplastics abundance (particles kg^−1^) by color in sea salt samples from stations S1 to S8.

**Figure 7:**
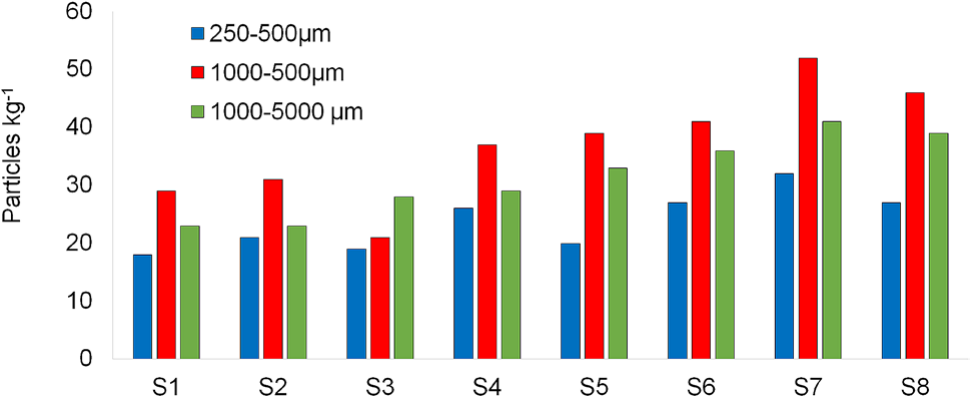
Microplastics abundance (particles kg^−1^) by size range in sea salt samples from stations S1 to S8.

The abundance of MP in salt samples by color and size range is presented in Figs. 6 and 7 and Table 1. The colors identified include white, blue, green, black, pink, transparent, and colorless. The distribution was: white (37%) > black (17%) > blue (15%) > green and transparent (10% each) > pink (6%) > colorless (5%). In terms of size, most particles were in the category 500–1000 µm, except for S3 (1000–5000 µm) (Table 1). The distribution of MP particles based on size range was: 500–1000 µm (40%) > 1000–5000 µm (34%) > 250–500 µm (26%). For salts from the Atlantic and the Pacific Ocean, originating from Brazil, the United Kingdom, and the USA, Kim et al.^12^ reported a higher abundance of MP in size range 100–1000 µm while sizes in the range 100–5000 µm were reported for salt samples from the Black Sea. Seth and Shriwastay^20^ found that 80% of fibers found in salt samples from the Indian Sea were smaller than 2000 μm in length. MP size range differences among the various studies are suggested to depend on the degree of weathering for a given environment^30^, different climatic conditions such as wind, rain, temperature, salinity, and waves influencing size range composition. Also, for runoff, rivers, and atmospheric fallout transportation, smaller MP size ranges can be expected to be associated with a longer range from the initial plastic sources^31–33^. Nevertheless, more detailed information about MP polymer/color features within the size ranges are needed to achieve stronger conclusions about potential long/short-range sources.

### MP polymer composition

**Figure 8:**
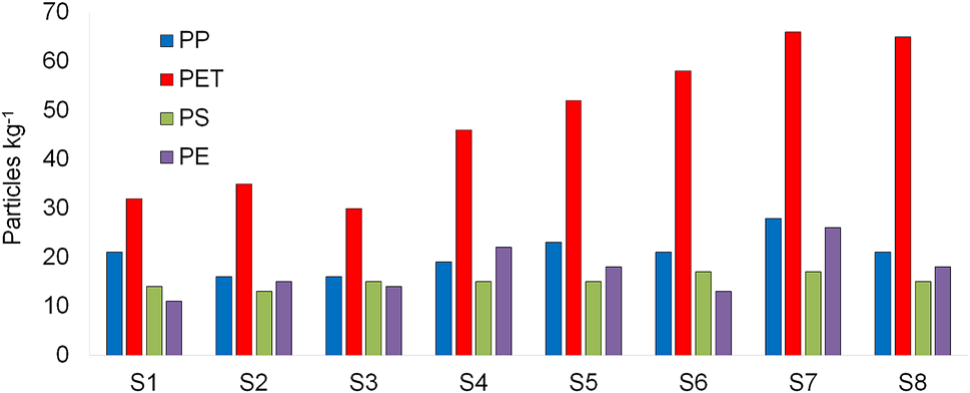
Microplastics abundances (particles kg^−1^) by polymer composition in sea salt samples from stations S1 to S8.

Four types of polymer, namely polypropylene (PP), polystyrene (PS), polyethylene (PE), and polyethylene terephthalate (PET), were identified with FT-MIR-NIR (Supplementary Figure S1). These results are in accordance with those reported for salt samples in other studies worldwide (Table 1). These polymer types are widely used in daily life products, packaging, single-use plastics, and clothes, contributing to plastic pollution worldwide^21^. PET presented the highest contribution at all sampling sites, at ~ 48%, whereas PS was found to be least, at ~ 15% (Fig. 8, Table 1). Iñiguez et al.^34^ also reported PET predominance (83.3%) in Spanish table salt samples. PET predominance could be explained by its high density (1.30 g cm^−3^), making particles prone to sedimentation during the salt crystallization process^19^. PE (0.94 g cm^−3^), PP (0.90 g cm^−3^), and PS (1.05 cm^−3^) presented lower or similar densities to seawater (~ 1.02 g cm^−3^), making these more prone to flotation and possible loss due to wind during desiccation.

### Risks assessment

**Figure 9:**
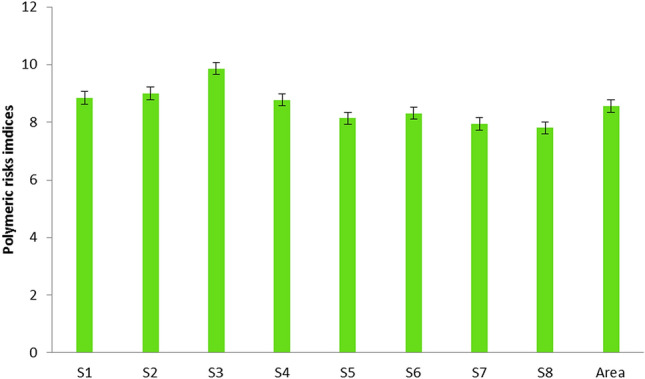
Polymeric risk indices for MP types in salts from stations S1 to S8.

During degradation, MP tends to emit monomers and different types of additives, these having the potential to cause harm to ecological systems and health^18, 35^. Results for the polymeric risks indices are presented in Fig. 9. According to polymer risk classification, all salts samples showed low risks, being similar to the entire study area. To date, none of the published studies have applied chemometric models in evaluating MP pollution in salts, posing difficulties when comparing our results. Information on the hazards of MP from ingestion to human health is still highly unclear. Other than exposure, the destiny and transit of ingested MP in the human body, including intestinal digestion and biliary discharge, have not been determined in previous research and remained largely unclear^36^. Some studies conducted impact assessments based on in vitro models^37,38^. However, whether the exposure concentrations used in such studies indicate the MP consumed and collected in humans is inconclusive. Previous studies found that toxicity, oxidative stress, and inflammation could result from MP exposure, including immune disruption and neurotoxicity effects, among others^39^. Therefore, an immediate effort is required to assess the health consequences of these MP when they reach the human body.

## Conclusions

Microplastics in salt have become a critical issue of environmental pollution and public health. The present study has provided the first report on MP contamination in coarse salt samples from Bangladesh. A total of eight salt pans along the Maheshkhali Channel of the Bay of Bengal were selected for this study, all samples showing the presence of MP concentrations in the range from 78 ± 9.33 to 137 ± 21.70 particles kg^−1^. Most MPs were white and in the size range 500–1000 µm. The predominant shapes were fragments and films (70%). Fourier transform mid-IR spectrum, and near-IR spectrum (FT-MIR-NIR) analysis registered the presence of polyethylene terephthalate (48%), polypropylene (20%), polyethylene (17%), and polystyrene (15%) in all samples. The plastic cover used for salt desiccation, urbanization including household effluents, local fisheries, industries, and tourism activities, were the primary potential plastic pollution sources. The results contribute to a better knowledge of MP presence in sea salts in Bangladesh and may help to prompt actions to reduce human exposure to MP in the future.

## Supplementary Information

Supplementary Information.
