# Supplementary material for: Microplastics pollution in salt pans from the Maheshkhali Channel, Bangladesh
Source: Sci Rep. 2021 Nov 30;11:23187. doi: 10.1038/s41598-021-02457-y (PMC8632905; doi:10.1038/s41598-021-02457-y)
Supplement: Supplementary file 1 — Supplementary Information. [file 41598_2021_2457_MOESM1_ESM.docx]

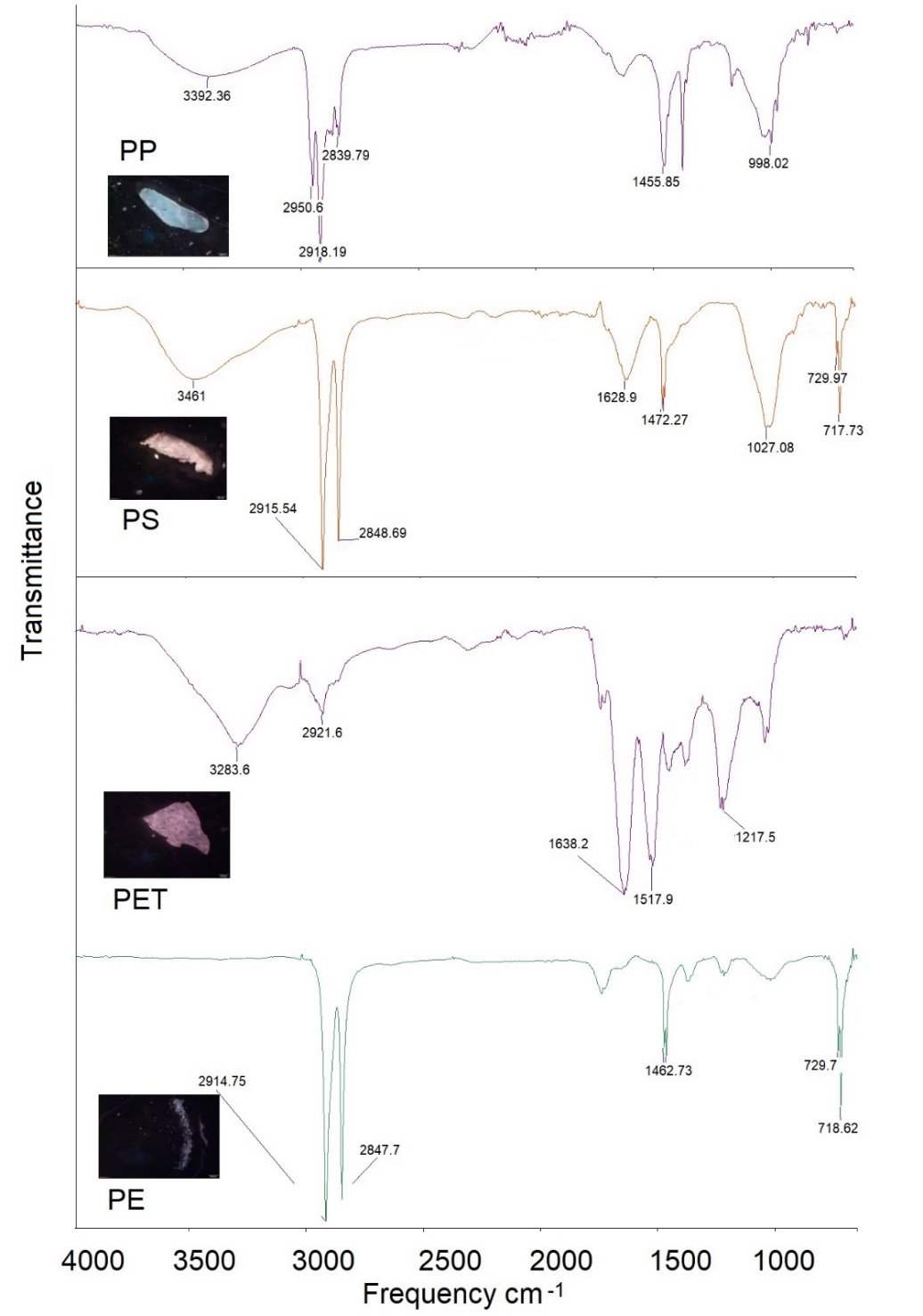


**Supplementary Figure S1.** Microplastics spectra obtained with FT-MIR-NIR for polypropylene (PP), polystyrene (PS), Polyethylene terephthalate (PET) and polyethylene (PE).
